# Dye-Modified, Sonochemically Obtained Nano-SnS_2_ as an Efficient Photocatalyst for Metanil Yellow Removal

**DOI:** 10.3390/ma16175774

**Published:** 2023-08-23

**Authors:** Grzegorz Matyszczak, Paweł Jóźwik, Magdalena Zybert, Albert Yedzikhanau, Krzysztof Krawczyk

**Affiliations:** 1 Department of Chemical Technology, Faculty of Chemistry, Warsaw University of Technology, Noakowskiego Street 3, 00-664 Warsaw, Poland; 2 Faculty of Advanced Technologies and Chemistry, Military University of Technology, Gen. Sylwester Kaliski Street 2, 00-908 Warsaw, Poland

**Keywords:** tin(IV) sulphide, nanopowder, sonochemistry, Phenol Red, bandgap, photocatalysis

## Abstract

We investigate the possibility of modification of SnS_2_ powder through sonochemical synthesis with the addition of an organic ligand. For that purpose, two organic dyes are used, Phenol Red and Anthraquinone Violet. All obtained powders are characterized using XRD, SEM, EDX, FT-IR, and UV-Vis investigations. Synthesized samples showed composition and structural properties typical for sonochemically synthesized SnS_2_. However, investigation with the Tauc method revealed that SnS_2_ powder modified with Phenol Red exhibits a significant shift in value of optical bandgap to 2.56 eV, while unmodified SnS_2_ shows an optical bandgap value of 2.42 eV. The modification of SnS_2_ powder with Anthraquinone Violet was unsuccessful. The obtained nanopowders were utilized as photocatalysts in the process of Metanil Yellow degradation, revealing that SnS_2_ modified with Phenol Red shows about 23% better performance than the unmodified one. The mean sonochemical efficiency of the performed synthesis is also estimated as 9.35 µg/W.

## 1. Introduction

The first structural report on tin(IV) sulphide is almost 100 years old; however, it is a very interesting and useful material according to many studies [1]. It is continuously investigated in a wide spectrum of practical applications, amongst which the most recent for standalone, composite, or combined in heterostructure SnS_2_ include electrocatalysis, gas sensors, humidity sensing, energy storage, and Li-ion batteries, solid-state extraction of antibiotics, photodetectors, and photocatalysis [2,3,4,5,6,7,8,9]. Tin selenides (SnSe, SnSe_2_), which are closely related to tin sulphides (SnS, SnS_2_), have many similar applications as well [10].

The above applications typically require materials in the form of nano- or microparticles, and thin films. There are many methods of obtaining such products, e.g., high-temperature solvent reaction, hot-injection method, solvothermal reaction, electrodeposition, etc. [11,12,13,14,15,16,17,18]. Another useful preparative method for the production of materials, which is easy to scale up, is sonochemical synthesis, which is a green method due to avoiding the use of toxic solvents and extreme conditions [19]. An additional advantage of this method is the possibility for wide modifications of synthesis conditions and combinations with other techniques, such as electrochemistry, allowing for better control of the properties of obtained particles [19,20,21]. As well as in the case of many materials, the synthesis of nano- and micropowders of SnS_2_ via the sonochemical route was demonstrated and thoroughly investigated, showing the broad dependence of the optical energy bandgap, morphology, and particle size on the used reagents and the applied experimental conditions [22,23,24].

Advanced materials have many applications, as mentioned earlier. One of them is photocatalysis. Particularly, inorganic materials may be utilized as photocatalysts in the degradation of organic compounds such as dyes and pharmaceuticals [25,26,27]. Examples of such materials are magnesium oxide (MgO) nanoparticles, the TiO_2_/CoFe_2_O_4_ nanocomposite, and the ternary TiO_2_/g-C_3_N_4_@Ag nanocomposite [25,26,27,28]. Many properties of photocatalysts may be tuned to increase their efficiency. In this context, one can mention the ability for light absorption, trapping, and recombination of the excited holes and electrons, the energy bandgap of the material, the presence of impurities, the size of particles, and more [29]. The energy bandgap determines what light (i.e., of what energy) is absorbed by the material [29]. The improvement of catalytic properties of photocatalysts may be achieved through dye functionalization (sensitization) which may affect the energy bandgap [30,31,32,33,34,35,36,37].

Theoretical and experimental investigations of monolayers, nanoparticles, surfaces, and clusters of distinct chemical compounds reveal the possibility of bandgap tuning via functionalization with ligands, such as chromophores or conjugated organic groups [38,39,40,41]. Synthesis of a nanopowder with a bandgap tuning ligand was demonstrated (for example) in the case of a high-temperature reaction in a solvent, but there are no reports on such an approach in sonochemical synthesis [42]. This is probably because it may yet be hampered by the possible modification or even destruction of molecules of organic ligands by radicals generated in the reaction medium as a result of the action of ultrasound radiation (e.g., water sonolysis) [43,44].

This study presents the investigation of the possibility for modification of SnS_2_ nanopowder with organic molecules in sonochemical synthesis. The attempt of modification is performed using two organic dyes—Phenol Red and Anthraquinone Violet. These dyes were chosen because they represent distinct dye types and colors on opposite sides of the visible light spectrum. The obtained products of the syntheses are characterized using X-ray powder diffraction, EDX spectroscopy, and FT-IR spectroscopy to confirm the presence of SnS_2_. The Tauc method is used to study the influence of functionalization on the optical bandgap of the synthesized materials, and SEM observations reveal the morphology of their powders. The obtained products are compared in terms of the efficiency of photocatalytic removal of Metanil Yellow under UV-C irradiation.

## 2. Materials and Methods

### 2.1. Materials and Reagents

**Figure 1:**
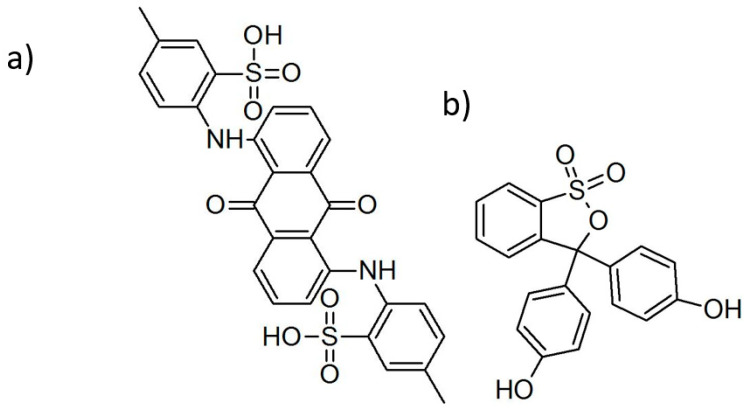
Molecular structure of (**a**) Anthraquinone Violet and (**b**) Phenol Red.

All chemicals used in this study were pure for analysis (producer: POCH—Polskie Odczynniki Chemiczne, Warsaw, Poland). For sonochemical syntheses, SnCl_4_∙5H_2_O and thioacetamide (TAA) were used as reagents, and Anthraquinone Violet (Figure 1a) and Phenol Red (Figure 1b) were used as modifying ligands. Ethanol was used as a solvent and for the purification of prepared suspensions. Tert-Butanol (99.5%, Acros Organics, Waltham, MA, USA) was used as a radicals scavenger.

### 2.2. Sonochemical Syntheses

The sonochemical syntheses were conducted in conical flasks of 50 mL volume in an ultrasonic cleaner (PS 10A) generating an ultrasound of 40 kHz frequency with a nominal power of ultrasounds at 60 W. The acoustic power determined calorimetrically was 27.9 W/L. The procedure for syntheses and purification of obtained powders was adopted from the Matyszczak et al. investigation [24].

In all syntheses, the following amounts of reagents were used: 701 mg of SnCl_4_∙5H_2_O, 376 mg of thioacetamide, and 20 mL of solvent. Additionally, 10 mg of relevant dye was added to the reaction mixture before sonication. All reagents were stirred for 10 min with a magnetic stirrer before the start of the reaction. The duration of sonication was 100 min. After that and before the purification, the open conical flasks with obtained powders suspended in the original reaction mixture were kept under laboratory hood for 3 days.

**Figure 2:**
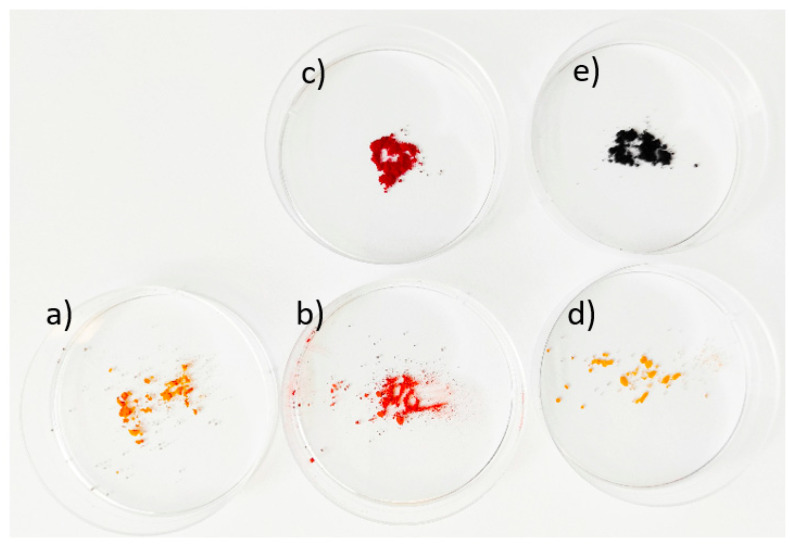
Photograph of synthesized powders and (for comparison) dyes: (**a**) unmodified SnS_2_, (**b**) SnS_2_ modified with Phenol Red, (**c**) Phenol Red, (**d**) SnS_2_ unmodified with Anthraquinone Violet (unsuccessful modification), (**e**) Anthraquinone Violet.

Photograph of dried powders (products of syntheses) and used dyes is presented in Figure 2.

### 2.3. Evaluation of Sonochemical Efficiency and Percentage Yield of the Synthesis Process

The percentage yield and the sonochemical efficiency of the synthesis process were calculated according to the two following equations:

(1) $$Y=\frac{m_{dry}}{m_{theoretical}}\cdot 100\%$$

(2) $$SE=\frac{m_{dry}}{P_{ultrasound}}$$

where

Y—percentage yield [%].

$m_{dry}$—mass of dried product [µg].

$m_{theoretical}$—theoretical mass of dried product calculated based on the taken amount of reagents assuming full overreaction [µg].

SE—sonochemical efficiency [µg/W].

$P_{ultrasound}$—the power of ultrasound determined calorimetrically [W].

Both values (*Y* and *SE*) are calculated in reference to the dried products which were obtained by drying purified suspensions in ethanol under a laboratory hood for 3 days.

### 2.4. Characterization of Products

The characterization of synthesized products was performed according to the procedure presented in the Matyszczak et al. study [24]. Powders were investigated using the following techniques: powder X-ray diffraction (PXRD) (RTG HZG-4 diffractometer, Siemens, Munich, Germany), scanning electron microscopy (SEM) (Quanta 3D FEG, FEI Thermo Fisher Scientific, Hillsboro, OR, USA) equipped with energy-dispersive X-ray spectroscopy (EDX) (EDAX, Gatan, Pleasanton, CA, USA), Fourier transform infrared spectroscopy (FT-IR) (NICOLET 6700 FT-IR spectrometer, Thermo Fisher Scientific, Waltham, MA, USA), and UV-Vis spectrophotometry (the Tauc method) (UV1600 spectrophotometer AOE Instruments, Shanghai, China).

The UV-Vis spectra were collected using suspensions in ethanol of synthesized powders, instead of suspensions in chloroform.

### 2.5. Photocatalytic Degradation of Metanil Yellow

**Figure 3:**
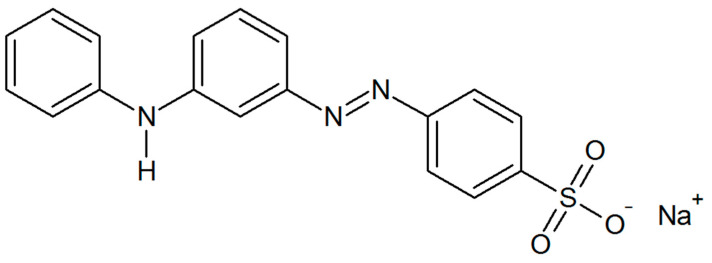
Molecular structure of Metanil Yellow azo-dye.

As a sample dye for photocatalytic removal, we used Metanil Yellow (Figure 3) which is highly toxic and widely used in the food industry [45,46,47,48]. The process was conducted in a glass beaker of 50 mL volume under ambient temperature and pressure. An amount of 20 mL of dye solution in distilled water was placed in the beaker equipped with a magnetic bar. Then, 20 mg of dried relevant SnS_2_ powder was used to make 10 mL of photocatalyst’s suspension in distilled water. The suspension was sonicated for 30 min. Such a suspension was then transferred quantitatively to the previously measured 20 mL of dye solution. The prepared mixture was then mixed with a magnetic stirrer with a speed of 600 rpm under dark conditions for 30 min to ensure the establishment of adsorption–desorption equilibrium, and then, the UV-C lamp (72 W) or UV-A lamp (10 W) was turned on for 150 min. After the process, the mixture was centrifuged (8000 rpm, 6 min) twice to obtain a clear dye solution, free of suspended catalyst and ready for absorbance measurement. The absorbance was measured at a wavelength of 440 nm using a UV-Vis spectrophotometer (UV1600 spectrophotometer AOE Instruments, Shanghai, China). Each experiment, including the blind test, was conducted 3 times.

Additional experiments were also conducted with the addition of 1 mL of t-BuOH as a scavenger of hydroxyl radicals. This amount of t-BuOH was added instead of 1 mL of distilled water during the preparation of the suspension of catalyst (i.e., 20 mg of catalyst was suspended in a mixture of 9 mL of distilled water and 1 mL of t-BuOH). Also, KI was used as a scavenger of positive holes in a concentration of 10 mM in the reaction mixture.

### 2.6. N_2_ Physisorption Measurements

The specific surface area and porosity of the samples were determined using the nitrogen physisorption method (ASAP2020 instrument, Micromeritics Instrument Co., Norcross, GA, USA). Before the experiments, each sample was degassed at 50 °C for 1 h and then at 90 °C for 4 h under vacuum. The results of measurements in the relative pressure range of p/p_0_ = 0.05–0.3 were approximated using the Brunauer–Emmett–Teller isotherm model and provided information about the specific surface area. The approximation of the measurement results in the relative pressure range of p/p_0_ = 0.01–1.0 using the Barrett–Joyner–Halenda isotherm model was applied to obtain information about the porosity and pore distribution of the samples.

## 3. Results and Discussion

**Figure 4:**
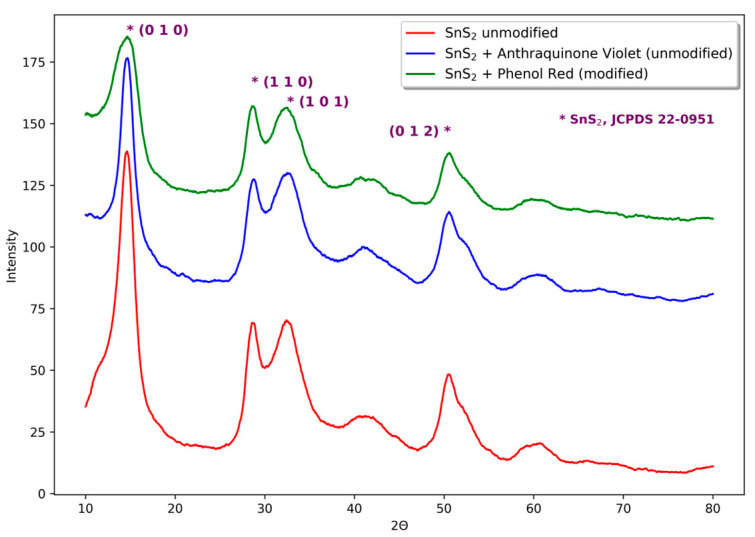
Powder X-ray diffraction patterns of synthesized products. (* denotes peaks linked to SnS_2_ phase)

As expected, all performed syntheses led to obtaining powders of SnS_2_. The dried products have a color typical for SnS_2_, while the product obtained with the addition of Phenol Red has its color changed to red (see Figure 2a,b,d). Powder X-ray diffraction investigation confirms the presence of low crystalline SnS_2_ (Figure 4). All three obtained products have similar diffraction patterns and exhibit the same reflexes. The broadening of reflexes indicates the presence of nanocrystallites. The mean particle sizes were calculated using the Scherrer equation:

(3) $$D=\frac{K\lambda }{\beta_{hkl}cos\theta }$$

where

*β_hkl_*—full-width at half maxima of diffraction peak in 2θ scale (in radians). 

*θ*—the diffraction angle (in radians).

*λ*—the wavelength of applied X-rays (in this study, *λ* = 1.54 Å).

*K*—the shape factor, assumed as 0.9 in this study.

*D*—the average crystallite size.

The crystals are ca. 4–5 nm (taking the value of shape factor 0.9). Due to the low crystallinity of obtained samples, such a result should be threatened only qualitatively.

**Figure 5:**
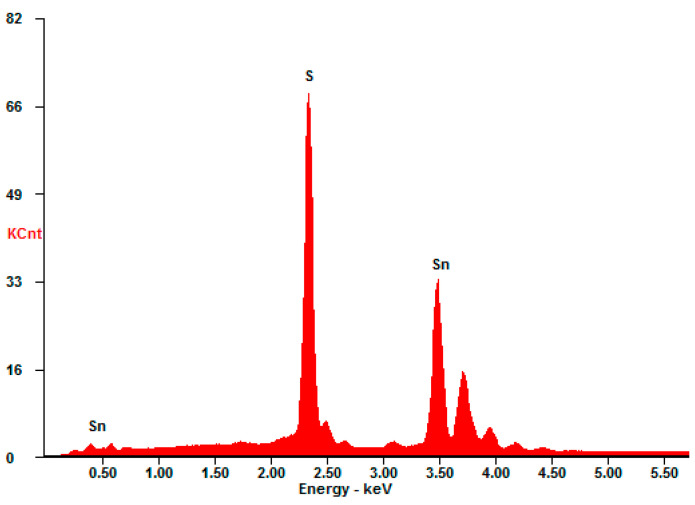
Typical EDX spectrum of synthesized powders.

Additionally, the presence of SnS_2_ in all synthesized samples is confirmed by energy-dispersive X-ray spectroscopy. For all samples, the atomic ratio of S to Sn is near 2:1 (exact ratios are in the range of 1.81–1.87). The sample EDX spectrum is presented in Figure 5.

Calculated percentage yields (Y) and sonochemical efficiencies (SE) for three different syntheses are almost the same. It seems that the dye addition does not affect the reaction course. The mean percentage yield estimated according to Equation (1) is 12.97% while the mean sonochemical efficiency calculated using Equation (2) is 9.35 µg/W. It should be emphasized that these values apply to the whole process, including the purification step, and to the reaction time of 100 min. Some losses are possible while removing the supernatant after centrifugations during the purification step. The calculated values of sonochemical efficiency and percentage yield may be used for process intensification.

Figure 2 shows that synthesis with the addition of Phenol Red led to a powder of SnS_2_ with changed color. This points to successful modification. However, the product of synthesis with the addition of Anthraquinone Violet remains unchanged. It seems that this dye is unable to modify SnS_2_ powder in the sonochemical synthesis in the applied conditions. Phenol Red has two groups potentially capable of bonding with the surface of SnS_2_: sulfone and hydroxyl groups. Anthraquinone Violet also contains sulfone groups in addition to amino and carbonyl groups. Taking into account that the modification was successful only in the case of Phenol Red, hypothetically, the hydroxyl groups are responsible for bonding with SnS_2_ particles.

**Figure 6:**
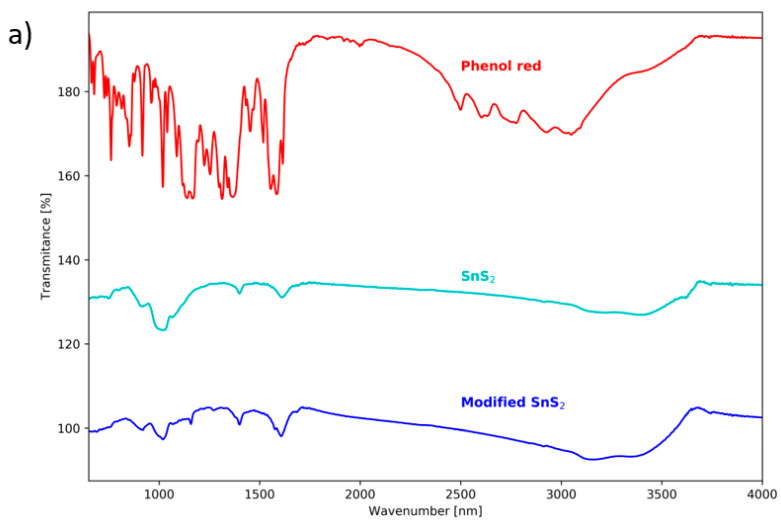

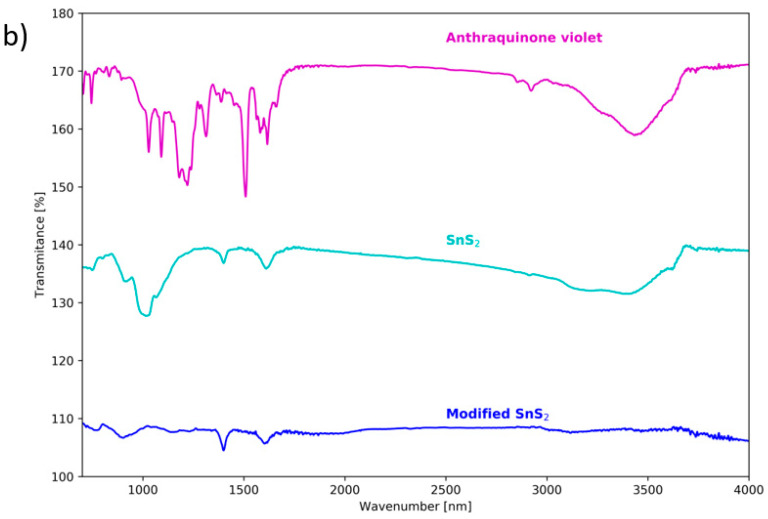
FT-IR spectra of used dyes and synthesized powders: (**a**) Phenol Red, unmodified SnS_2_, SnS_2_ synthesized with the addition of Phenol Red, (**b**) Anthraquinone Violet, unmodified SnS_2_, SnS_2_ synthesized with the addition of Anthraquinone Violet.

To further investigate the obtained powders and to check for the occurrence of dye molecules in them, FT-IR spectroscopy is applied. FT-IR spectra of synthesized SnS_2_ powders are in agreement with that reported previously (Figure 6) [24]. As Figure 6a shows, the Phenol Red in the modified SnS_2_ powder is present in an amount that is undetectable by FT-IR spectroscopy.

**Figure 7:**
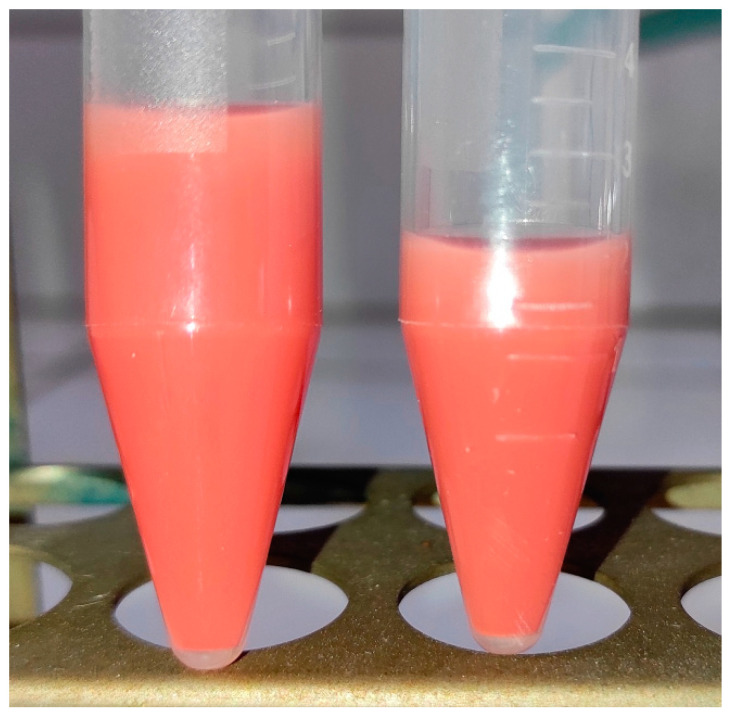
Photograph of suspensions in ethanol of SnS_2_ powder modified with Phenol Red before (**left**) and after (**right**) several centrifugations (each time adding a portion of fresh ethanol).

Additionally, the modification by Phenol Red is stable, because after several centrifugations (each time removing the supernatant and adding fresh ethanol), the color of the suspension of powder remains unchanged (Figure 7).

**Figure 8:**
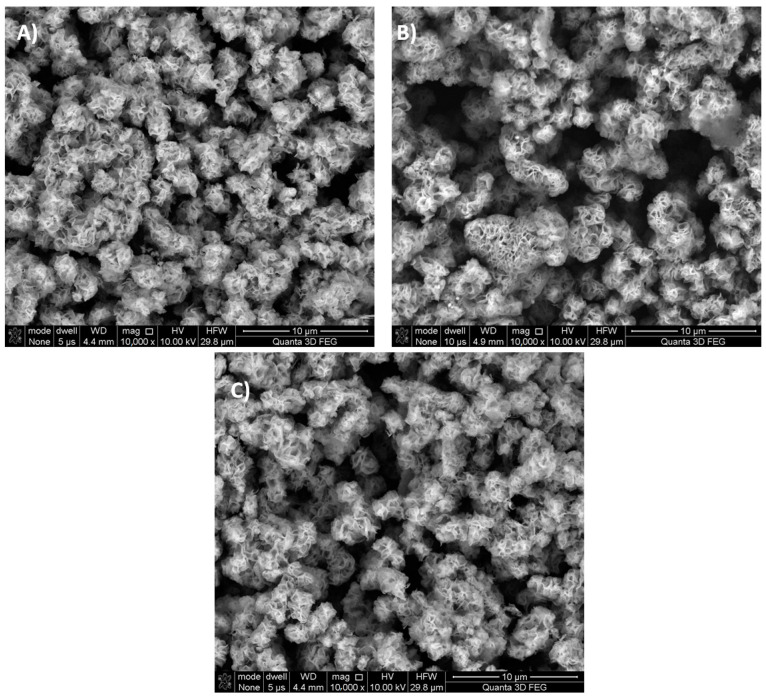
Scanning electron microscopy images of synthesized powders, recorded using back-scattered electrons. (**A**) SnS_2_ synthesized without organic additives (unmodified), (**B**) SnS_2_ synthesized with the addition of Phenol Red, and (**C**) SnS_2_ synthesized with the addition of Anthraquinone Violet.

**Figure 9:**
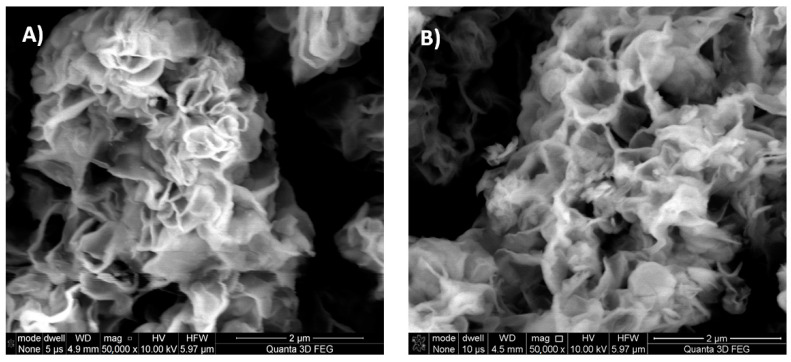
More scanning electron microscopy images of synthesized powders, recorded using back-scattered electrons with greater magnitude. (**A**) SnS_2_ synthesized with the addition of Phenol Red, (**B**) SnS_2_ synthesized without organic additives (unmodified).

Scanning electron microscopy investigation reveals that all synthesized powders, despite the addition of organic additives, share similar flower-like morphology, typical for SnS_2_ synthesized sonochemically [24] (Figure 8 and Figure 9). Also, there is no significant variation in the size of the particles. Apparently, due to the flower-like morphology, the particles would have the ability to lock the dye molecules inside them. However, this mechanism is not responsible for the modification of powder with Phenol Red, as according to such mechanism Anthraquinone Violet should also be incorporated into the powder changing its color, which is not observed.

SEM images of greater magnitudes (Figure 9) show that prepared SnS_2_ powders have elongated structures (thread-like) that have dimensions much less than 1 μm and lie in the nanorange.

**Figure 10:**
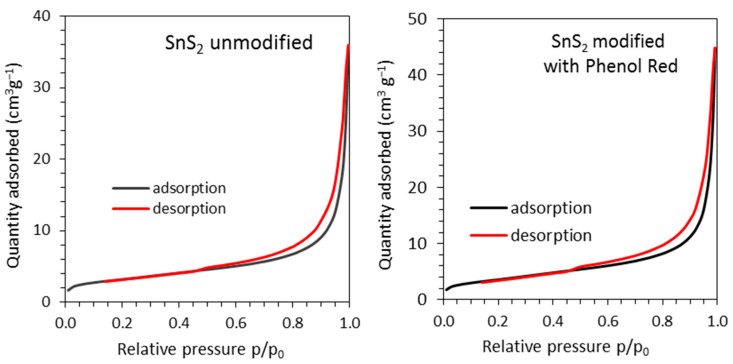
Nitrogen adsorption–desorption isotherms for the studied samples.

**Figure 11:**
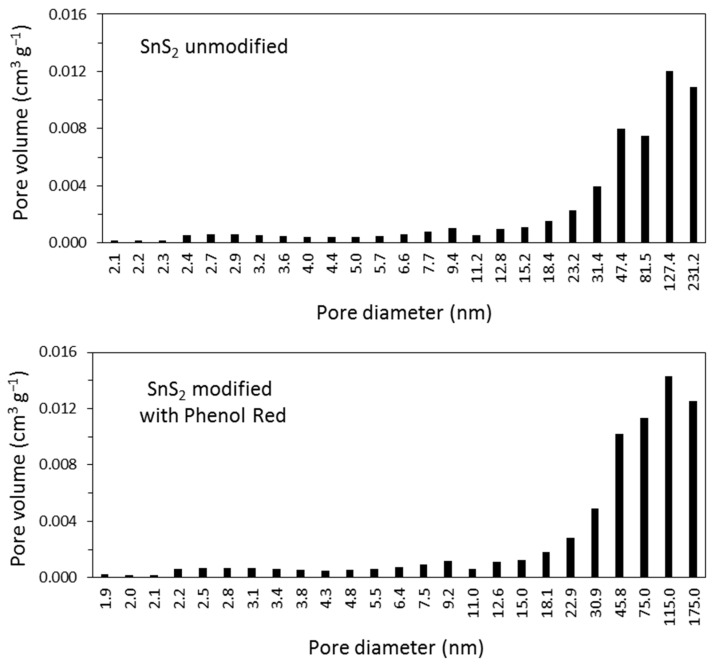
Pore volume distribution for the studied samples.

According to the N_2_ physisorption measurements, there were no significant differences in the textural properties of the prepared materials. The specific surface area and total pore volume were similar for both samples (Table 1). As shown in Figure 10, the materials’ low porosity was associated with a small number of mesopores and macropores, the presence of which was confirmed by the shape of N_2_ adsorption–desorption curves. Type IV isotherms with H3 hysteresis loop were observed for both materials. According to the IUPAC classification [49], this type of loop can be given by nonrigid aggregates of plate-like particles. Also, it may be connected with macropores not completely filled with pore condensate. The presence of both types of pores (mesopores and macropores) was also confirmed by the pore volume distribution presented in Figure 11.

**Figure 12:**
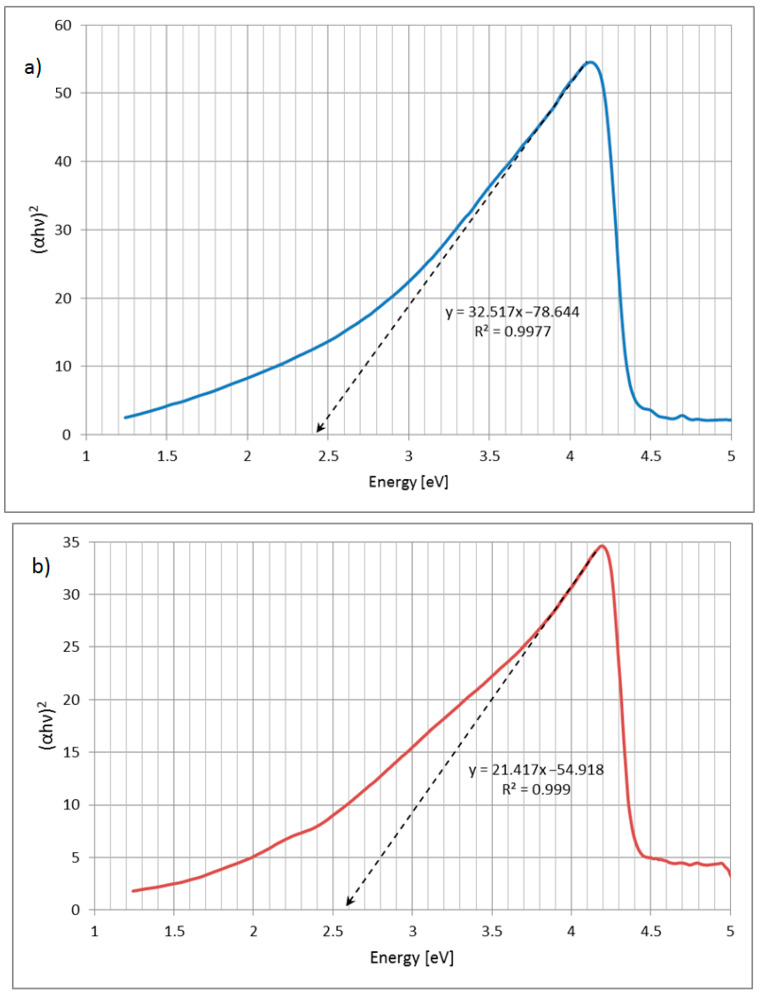
Tauc plots of the suspension in ethanol of SnS_2_: (**a**) unmodified; (**b**) modified with Phenol Red.

The optical bandgap of unmodified SnS_2_ determined using the Tauc method is 2.42 eV (Figure 12a). Such a value is typical for SnS_2_ [50]. Interestingly, the same method implies that SnS_2_ powder modified with Phenol Red has its optical bandgap significantly shifted to 2.56 eV (Figure 12b).

UV experiments proved the photocatalytic properties of both unmodified and modified SnS_2_. The dye solution without added catalyst irradiated with UV-C light for 150 min under dark conditions did not exhibit decolorization (blind test). For both catalysts, the experiment was conducted 3 times and the decrease in absorbance was converted (using the calibration curve and the reaction mixture volume) to a decrease in the amount of dye (in micrograms). Photocatalytic activities of the investigated powders were calculated according to the following equation (Equation (4)):

(4) $$PA=\frac{m_{dye}}{m_{catalyst}}$$

where

PA—photocatalytic activity [µg/mg].

$m_{dye}$—a mass of dye degraded during the photocatalytic process [µg].

$m_{catalyst}$—a mass of catalyst used in an experiment [mg] (20 mg in this study).

**Figure 13:**
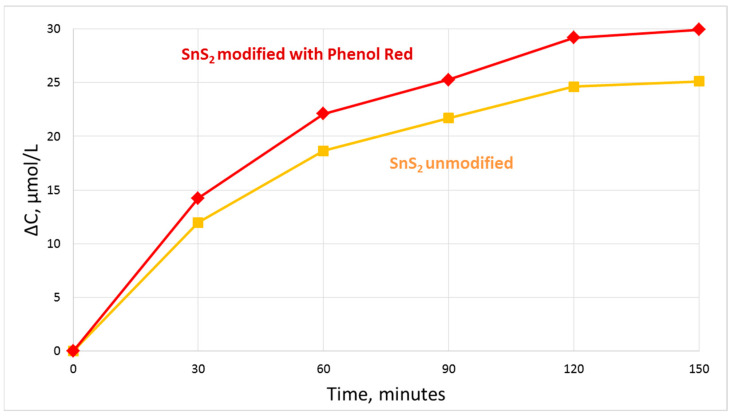
Decrease in concentration of azo-dye Metanil Yellow in the photocatalytic degradation process with usage of SnS_2_ modified with Phenol Red as photocatalyst in the function of process duration (squares—data for unmodified SnS2, rhombus—data for SnS2 modified with Phenol Red).

The mean photocatalytic activity of unmodified SnS_2_ is 13.73 µg of dye per 1 mg of catalyst. In the case of SnS_2_ powder modified with Phenol Red, the mean photocatalytic activity is 16.86 µg of dye per 1 mg of catalyst. These values are the results of experiments with the UV-C lamp of power 72 W. The Phenol Red-modified SnS_2_ powder has about 23% greater photocatalytic activity than the unmodified one which is apparently caused by dye modification. The kinetic study of degradation of Metanil Yellow by SnS_2_ photocatalysts synthesized in this study under UV-C lamp (72 W) shows that the decrease in the concentration of dye approaches asymptotic value at the time in a range ca. 120–150 min (Figure 13). Under the UV-A lamp of power 10 W, the catalytic activity of modified SnS_2_ was only 2.76 µg of dye per 1 mg of catalyst, which is ca. 6 times less than in the case of UV-C lamp of power 72 W.

**Figure 14:**
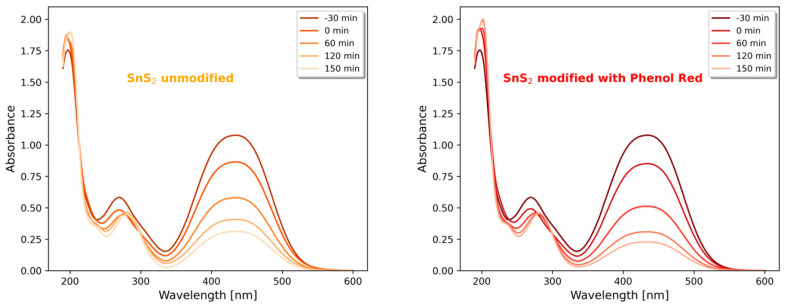
The raw UV-Vis absorption data during photocatalytic experiments (UV-C lamp 72 W) using unmodified SnS_2_ (**left**) and SnS_2_ modified with Phenol Red (**right**).

The UV-Vis spectra measured during the photocatalytic processes show the decrease in the main peak of absorption in the visible region, near 440 nm (Figure 14). The decrease is greater in the case where SnS_2_ modified with Phenol Red is used as a photocatalyst. Both materials also adsorb the degraded dye in similar amounts. The main peak near 440 nm is related to the azo group so the degradation of Metanil Yellow in the conducted experiments goes through the oxidation of the -N=N- fragment. In the UV region, two peaks may be seen, near 197 nm and 270 nm, which are related to the benzene rings present in the molecule of Metanil Yellow. These peaks slightly increase (197 nm) and decrease (270 nm) during the degradation. Moreover, they change their position from 197 nm to 201 nm and 270 nm to 281 nm, which is likely caused by the formation of various intermediate compounds with molecular structures based on Metanil Yellow but changed due to the action of hydroxyl radicals.

There are not many studies on the photocatalytic degradation of Metanil Yellow; however, it was previously reported that a catalyst consisting of TiO_2_ immobilized into polyvinyl alcohol/acrylic acid microgels showed photocatalytic activity on a level of 0.4–0.9 µg of dye per 1 mg of catalyst, which is far less than the results presented in this study [51]. The estimated values of the photocatalytic activity of synthesized powders may be used for the intensification of the photodegradation process.

Additionally, experiments conducted with the addition of tert-Butanol to the reaction mixture showed no decolorization. As tert-Butanol is a scavenger of hydroxyl radicals ·OH, it is concluded that the photocatalytic processes conducted in this study are generally mediated by ·OH radicals [52,53,54]. The photocatalytic degradation of dyes typically occurs via oxidation by holes and/or hydroxyl radicals so our results are in agreement with the findings of other researchers [53,54]. The experiments with KI scavenger showed a ca. 31% decrease in decolorization for both photocatalysts, which additionally proves that the process is mediated mainly by hydroxyl radicals. The mechanism of the photocatalytic process is proposed below:

– the photocatalyst absorbs the photon and is excited, then generates a pair of positive hole and negative electron:
SnS_2_ + hν → SnS_2_ + e^−^ + h^+^
– the hole reacts with a molecule of water generating hydroxyl radicals:
  h^+^ + H_2_O → H^+^ + ·OH
– the hydroxyl radicals attack the dye molecules and degrade (oxidize) them in many consecutive steps:
  Dye molecule + x ·OH →→→ y CO_2_ + z H_2_O

The ·OH radicals are the main intermediates for both catalysts (modified and unmodified); thus, the explanation of increased photocatalytic activity cannot be provided based on the mechanism of the process. The modified SnS_2_ has a larger bandgap so it should be harder to excite it with light; however, it shows better efficiency than the unmodified one. This could be due to two potential reasons. Firstly, the modified SnS_2_ may exhibit a much greater light absorption coefficient, absorbing more light than the unmodified SnS_2_ and generating more hole–electron pairs at the same time. Secondly, the presence of Phenol Red molecules on the surface of the photocatalyst may diminish the recombination of hole and electron which leads to a greater production of hydroxyl radicals.

## 4. Conclusions

We presented a novel method of modification of the optical properties of SnS_2_ obtained in the sonochemical synthesis. We showed that SnS_2_ may be modified with Phenol Red, while Anthraquinone Violet does not exhibit such behavior. Moreover, such modification is stable and does not change the structural or morphological properties of SnS_2_ powder. At the same time, it allows for a significant change in the value of the optical bandgap. This result may be used for bandgap tuning in a wide spectrum of applications, e.g., for solar energy harvesting and (as demonstrated in this study) photocatalysis. The values of sonochemical efficiency and photocatalytic activity presented in this study allow for intensification of the synthesis and photodegradation processes. Scavenging experiments showed that the process is ·OH mediated. The photocatalytic activity of the studied catalysts was much better than the one reported in the literature for the degradation of Metanil Yellow.

## Figures and Tables

**Table 1:** Textural parameters of the studied samples.

Sample	Specific Surface Area (m^2^ g^−1^)	Total Pore Volume (cm^3^ g^−1^)
SnS_2_ unmodified	11.4	0.056
SnS_2_ modified with Phenol Red	13.1	0.070

## Data Availability

The raw/processed data required to reproduce these findings cannot be shared at this time due to technical or time limitations.
